# Clinical significance of metabolic quantification for retinal nonperfusion in diabetic retinopathy

**DOI:** 10.1038/s41598-022-13439-z

**Published:** 2022-06-04

**Authors:** Areum Jeong, Xue Yao, Jano van Hemert, Min Sagong

**Affiliations:** 1 Department of Ophthalmology, Yeungnam University College of Medicine, #170 Hyunchungro, Nam-gu, Daegu, 42415 South Korea; 2 Yeungnam Eye Center, Yeungnam University Hospital, Daegu, South Korea; 3 Optos PLC, Dunfermline, UK

**Keywords:** Retinal diseases, Diabetes complications

## Abstract

Diabetic retinopathy (DR) is characterized by microvascular changes including ischemia. Degradation and metabolic changes of various retinal cells occur during ischemia. Ischemic region containing more cells will lead to greater metabolic impairment. We analyzed the non-perfusion region (NPR) by integrating histologic mapping with ultra-widefield fluorescein angiography (UWF FA) images. We also investigated the correlations of the weighted ischemic index (ISI) considering the regional distribution of retinal cells with cytokines, macular edema (ME), and neovascularization (NV). In this study, 32 patients with treatment-naïve DR and 21 age-matched control participants were included. The difference between the non-weighted and weighted ISI of NPR with leakage was greatest at the posterior region. The weighted ISI of NPR with leakage was correlated with MCP-1, IL-8, IL-6, PlGF, and VEGF-A levels, while the non-weighted ISI of NPR with leakage was correlated with IL-8 and IL-6 levels. The presence of baseline ME or NV in patients with DR was associated with the weighted ISI, with a stronger association when cones and rods were weighted. The weighted ISI reflecting both metabolic activity and cell distribution demonstrated a better correlation with clinical features and was more valuable in NPR with leakage than non-weighted ISI, which previous studies conventionally used.

## Introduction

Diabetic retinopathy (DR) is the leading cause of preventable visual impairment in the working-age population^1^. DR is characterized by microvascular pathologies, such as capillary basement membrane thickening, pericyte loss, capillary occlusion, and acellularity^2^. These ischemic changes have important roles in the development of diabetic macular edema (DME) and neovascularization (NV), promoting the production of various cytokines such as vascular endothelial growth factor (VEGF)^3^. Several studies using ultra-widefield (UWF) fluorescein angiography (FA) have demonstrated that the nonperfusion region (NPR) is related to the presence of diabetic macular edema (DME) and neovascularization (NV)^4–10^. However, there wasn’t linear correlation between them.

Ischemic insult causes cumulative retinal cell loss and dysfunction, as well as consequent alteration of metabolism at the cellular level. Several studies using electroretinography (ERG) and immunohistology showed destruction of retinal structure, including ganglion cells, amacrine cells, and even photoreceptors in ischemic condition^11–13^. Photoreceptors are known to play a major role in metabolism, contributing to metabolic homeostasis and retinal viability. Moreover, alteration of photoreceptor metabolism regulates pathologic angiogenesis by controlling angiogenic and inflammatory factors^14^. Previous studies have shown that deep capillary plexus loss is more prominent in eyes with increasing severity of DR and is correlated with photoreceptor integrity such as ellipsoid zone and external limiting membrane integrity^15^.

Recent advances in UWF applications and software offer reliable and accurate quantification. And it has been possible to integrate UWF images with topographic map of photoreceptors and ganglion cells provided by histologic studies^16,17^. Through this method, not only the ischemic region, but also the distribution of retinal cells reflecting metabolic activity could be considered.

Therefore, we analyzed the retinal ischemic area by integrating histologic mapping with UWF FA images in DR. We also investigated the correlations of the weighted index considering the regional distribution of retinal cells with various cytokines, ME, and NV.

## Results

### Demographic characteristics

The study included 32 eyes with severe non-proliferative DR (9 eyes) or proliferative DR (23 eyes). All patients with DR had type 2 diabetes and the mean diabetes duration was 7.8 ± 6.2 years. The mean age was 63.6 ± 10.2 years and 13 patients (40.6%) were male. The mean BCVA was 0.56 ± 0.32 logMAR, mean spherical equivalent was − 0.07 ± 0.91 diopters, and mean CMT was 447.3 ± 135.2 μm. ME was observed in 27 eyes (84.4%) and NV was found in 23 eyes (71.9%). The 21 controls (38.1% male) had a mean age of 64.3 ± 7.9 years, mean spherical equivalent of − 0.32 ± 0.93 diopters, and mean CMT of 263.9 ± 13.6 μm. There were no significant differences between the two groups in terms of age, gender, or spherical equivalent (Table 1).

**Table 1:** Patient characteristics, demographics, clinical features, and cytokine levels.

Demographic features	DR (n = 32)	Control (n = 21)	*p* value
Age, years; mean ± SD	63.6 ± 10.2	64.3 ± 7.9	0.860*
**Gender, n (%)**			0.783^†^
Male	13 (40.6)	8 (38.1)	
Female	19 (59.4)	13 (61.9)	
**DR severity, n (%)**			
Mild NPDR	0		
Moderate NPDR	0		
Severe NPDR	9 (28.1)		
PDR	23 (71.9)		
Diabetes duration, years; mean ± SD	7.8 ± 6.2		
Spherical equivalent, diopters; mean ± SD	− 0.07 ± 0.91	− 0.32 ± 0.93	0.087*
BCVA, logMAR; mean ± SD	0.56 ± 0.32	0.35 ± 0.37	0.025*
CMT, μm; mean ± SD	447.3 ± 135.2	263.9 ± 13.6	< 0.001*
Presence of ME	27 (84.4)		
Presence of NV	23 (71.9)		
**Baseline measurements using UWF FA; mean ± SD**			
Non-weighted ISI	0.49 ± 0.20		
Weighted ISI	0.41 ± 0.17		
**Baseline cytokine levels, pg/mL; mean ± SD**			
Ang-1	122.91 ± 437.21	35.08 ± 36.59	0.041*
Ang-2	412.21 ± 1417.98	27.53 ± 36.88	0.029*
MCP-1	942.49 ± 1080.65	646.55 ± 247.83	0.002*
IL-8	56.70 ± 158.77	12.05 ± 10.23	< 0.001*
IL-6	50.21 ± 167.23	3.98 ± 5.74	< 0.001
PDGF-AA	61.62 ± 183.89	24.94 ± 7.92	0.186
PlGF	32.95 ± 120.27	0.71 ± 0.50	0.012*
VEGF-A	244.41 ± 323.82	57.64 ± 22.48	< 0.001*

*BCVA* best-corrected visual acuity, *DR* diabetic retinopathy, *CMT* central macular thickness, *ISI* ischemic index, *logMAR* logarithm of the minimum angle of resolution, *ME* macular edema, *NPDR* non-proliferative diabetic retinopathy, *NV* neovascularization, *PDR* proliferative diabetic retinopathy, *SD* standard deviation,

*Mann–Whitney U test.

^†^Chi-square test.

Patients with DR had significantly higher levels of Ang-1 (*p* = 0.041), Ang-2 (*p* = 0.029), MCP-1 (*p* = 0.002), IL-8 (*p* < 0.001), IL-6 (*p* < 0.001), PlGF (*p* = 0.012), and VEGF-A (*p* < 0.001) than controls. The PDGF-AA levels did not differ statistically between two groups (*p* = 0.186). (Table 1).

### Distributions of weighted and non-weighted ISI of NPR with leakage among retinal zones

**Figure 1:**
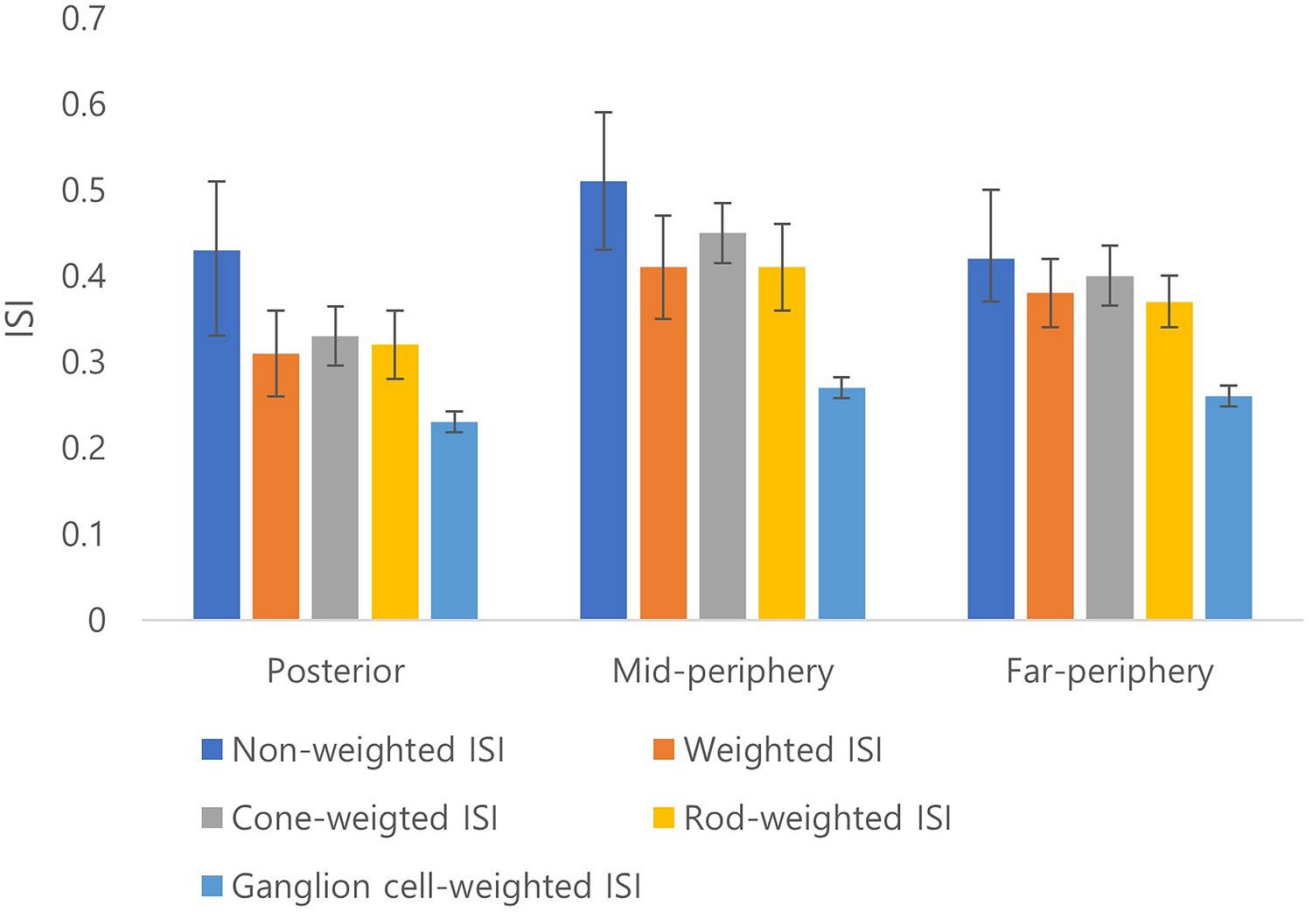
The distributions of weighted and non-weighted ischemic index (ISI) of nonperfusion region (NPR) with leakage among retinal zones.

For the entire retina, the mean non-weighted ISI was 0.49 ± 0.20, and the weighted ISI was 0.41 ± 0.17. The mean values of non-weighted ISI within the specific regions were as follows: posterior, 0.43 ± 0.10; mid-periphery, 0.51 ± 0.08; and far-periphery, 0.42 ± 0.06. The mean values of weighted ISI within the specific regions were as follows: posterior, 0.31 ± 0.05; mid-periphery, 0.41 ± 0.06; and far periphery, 0.38 ± 0.04. The difference between the non-weighted ISI and weighted ISI was greatest in the posterior region (p = 0.001). When analyzed according to cell type, cone weighted index and rod weighted index among different retinal zones were significantly different (p < 0.001), whereas the ganglion cell weighted ISI was not different (Fig. 1).

### Correlations of weighted and non-weighted ISI with BCVA, CMT, and cytokine levels

**Figure 2:**
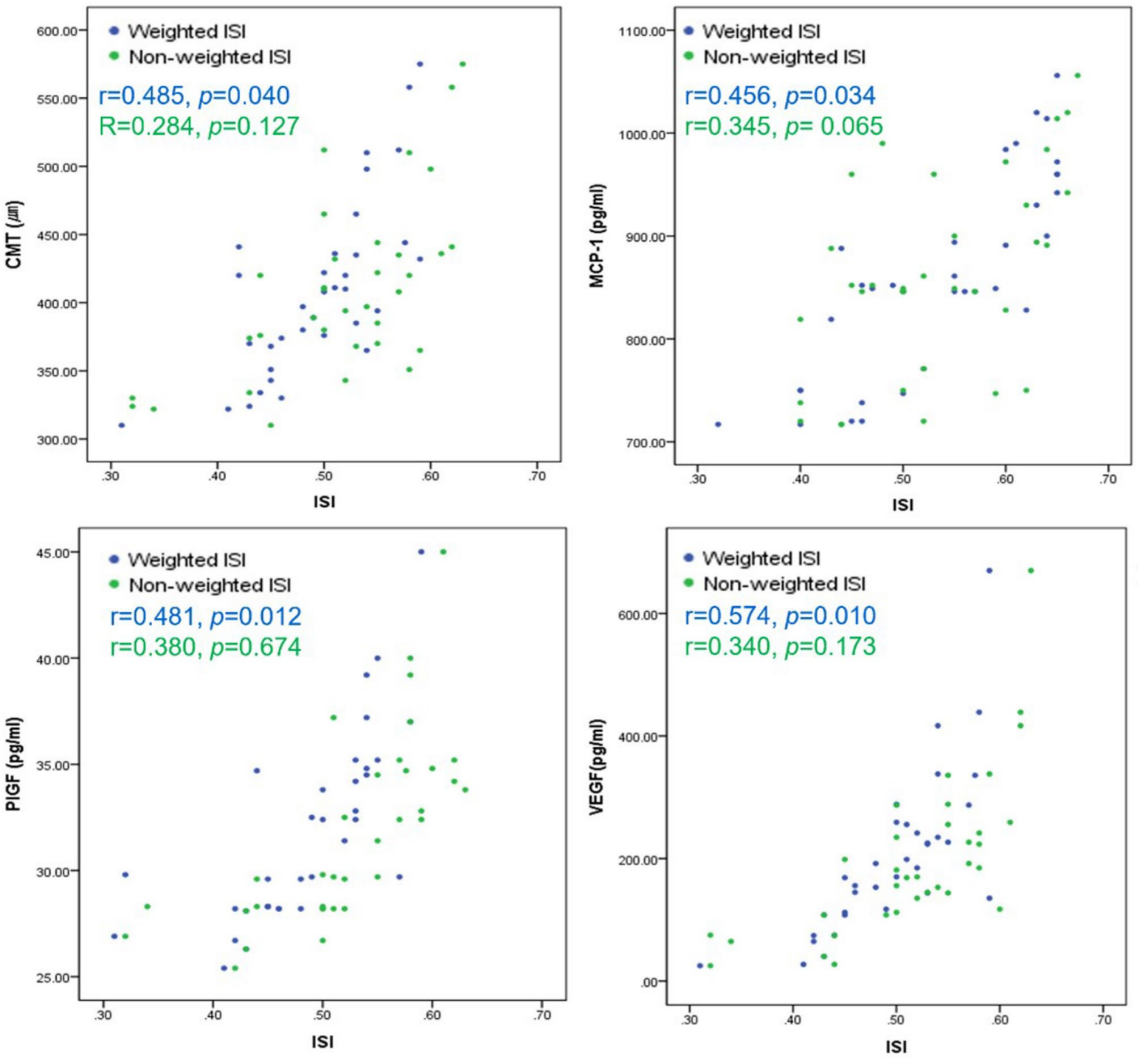
Correlations of the ischemic index (ISI) of NPR with leakage with the central macular thickness (CMT) and aqueous cytokine levels. While the weighted ISI of NPR with leakage had a significant positive correlation with CMT, levels of monocyte chemoattractant protein (MCP)-1, placental growth factor (PlGF), and vascular endothelial growth factor (VEGF)-A, the non-weighted ISI of NPR with leakage showed no significant correlation with them.

The non-weighted ISI of NPR with leakage was correlated with the levels of IL-8 (r = 0.284, *p* = 0.041) and IL-6 (r = 0.218, *p* = 0.039). The weighted ISI of NPR with leakage was correlated with CMT (r = 0.485, *p* = 0.040) and the levels of MCP-1 (r = 0.456, *p* = 0.034), IL-8 (r = 0.448, *p* = 0.010), IL-6 (r = 0.336, *p* = 0.018), PlGF (r = 0.481, *p* = 0.012), and VEGF-A (r = 0.574, *p* = 0.010). In the analysis according to cell type, the cone weighted ISI and rod weighted ISI of NPR with leakage were correlated with BCVA, CMT, and the levels of MCP-1, IL-8, IL-6, PlGF, and VEGF-A (Fig. 2). However, the ganglion cell weighted ISI of NPR with leakage did not correlate with any other variable.

The non-weighted ISI of NPR without leakage was not correlated with BCVA, CMT, or any cytokine levels. The weighted ISI of NPR without leakage was correlated with the levels of IL-8 (r = 0.282, *p* = 0.038) and IL-6 (r = 0.273, *p* = 0.020). In the analysis according to cell type, there were no correlations between the ganglion cell weighted ISI of NPR without leakage and other variables, whereas the cone weighted ISI and rod weighted ISI of NPR without leakage were correlated with the levels of IL-8 and IL-6 (Table 2).

**Table 2:** Correlations of weighted and non-weighted ISI with visual acuity, central macular thickness, and levels of cytokines.

	BCVA		CMT		Ang-1		Ang-2		MCP-1		IL-8		IL-6		PDGF-AA		PlGF		VEGF-A	
	R	*P*	R	*P*	R	*P*	R	*P*	R	*P*	R	*P*	R	*P*	R	*P*	R	*P*	R	*P*
**Nonperfusion region with leakage**																				
Non-weighted	0.242	0.680	0.284	0.127	− 0.052	0.468	0.068	0.321	0.345	0.065	0.284	0.041*	0.218	0.039*	0.045	0.874	0.380	0.674	0.340	0.173
Weighted ISI	0.319	0.472	0.485	0.040*	0.179	0.352	0.372	0.051	0.456	0.034*	0.448	0.010*	0.336	0.018*	0.312	0.287	0.481	0.012*	0.574	0.010*
Cone	0.428	0.011*	0.447	0.038*	− 0.062	0.667	0.148	0.144	0.431	0.041*	0.369	0.020*	0.304	0.034*	0.287	0.191	0.429	0.040*	0.519	0.038*
Rod	0.226	0.020*	0.421	0.025*	0.146	0.479	0.279	0.056	0.470	0.023*	0.453	0.015*	0.413	0.029*	0.349	0.078	0.478	0.008*	0.542	0.012*
Ganglion cell	0.116	0.132	0.163	0.411	0.532	0.243	0.367	0.068	0.242	0.062	0.217	0.124	0.147	0.104	0.227	0.412	0.347	0.054	0.223	0.060
**Nonperfusion region without leakage**																				
Non-weighted	0.121	0.655	0.175	0.346	− 0.062	0.436	− 0.075	0.524	− 0.068	0.512	0.255	0.063	0.195	0.057	− 0.012	0.823	− 0.064	0.453	− 0.120	0.487
Weighted ISI	0.124	0.438	0.136	0.464	− 0.126	0.418	− 0.032	0.893	− 0.052	0.776	0.282	0.038*	0.273	0.020*	− 0.020	0.673	0.057	0.566	0.023	0.840
Cone	0.156	0.345	0.140	0.982	− 0.174	0.325	− 0.115	0.562	0.061	0.715	0.262	0.041*	0.263	0.010*	0.325	0.434	0.113	0.693	0.210	0.462
Rod	0.085	0.636	0.184	0.770	− 0.112	0.513	0.043	0.648	0.043	0.766	0.303	0.030*	0.324	0.026*	− 0.132	0.731	0.179	0.284	0.078	0.645
Ganglion cell	0.047	0.647	0.097	0.612	− 0.131	0.332	0.074	0.577	0.052	0.734	0.145	0.632	− 0.179	0.346	− 0.064	0.695	0.086	0.513	0.034	0.742

P-values were calculated using Spearman correlation.

*BCVA* best-corrected visual acuity, *CMT* central macular thickness, *ISI* ischemic index, *R* Spearman correlation coefficient.

*P < 0.05.

### Associations of baseline characteristics with the weighted and non-weighted ISI of NPR with leakage

In the multiple linear regression analysis, ME at baseline was associated with the weighted ISI of NPR with leakage (difference, 3.80, *p* = 0.045), and was positively associated with the cone weighted ISI of NPR with leakage (difference, 3.64, *p* = 0.036) and the rod weighted ISI of NPR with leakage (difference, 4.19, *p* = 0.029).

NV at baseline showed a strong association with the weighted ISI of NPR with leakage (difference, 8.70, *p* = 0.044), and was positively associated with the cone weighted ISI of NPR with leakage (difference, 10.48, *p* = 0.035) and the rod weighted ISI of NPR with leakage (difference, 14.82, *p* = 0.012) (Table 3).

**Table 3:** Associations of baseline characteristics with weighted and non-weighted ISI of nonperfusion with leakage.

Confounding factor	ISI	Coefficient	*P* value
BCVA (logMAR)	Non-weighted ISI	2.64	0.481
	**Weighted ISI**		
	Total	3.29	0.313
	Cone	2.32	0.264
	Rod	4.45	0.153
	Ganglion cell	-0.18	0.821
ME at baseline (yes/no)	Non-weighted ISI	1.59	0.142
	**Weighted ISI**		
	Total	3.80	0.045
	Cone	3.64	0.036
	Rod	4.19	0.029
	Ganglion cell	0.09	0.085
NV at baseline (yes/no)	Non-weighted ISI	4.77	0.127
	**Weighted ISI**		
	Total	8.70	0.044
	Cone	10.48	0.035
	Rod	14.82	0.012
	Ganglion cell	1.50	0.117

P-values were calculated using multiple linear regression analysis.

*P < 0.05.

## Discussion

This study developed topographic histology mapping of photoreceptor and ganglion cell densities onto the UWF FA images. This new method allowed the measurement of cell counts of pixels correlated with metabolic activity across the retina. Retinal ischemia causes dysfunction and death of neuronal cells including photoreceptors and ganglion cells, by glial cell activation and immunomodulatory cytokine release^19–21^. In addition, various retinal cells such as Müller cells, lymph nodes, endothelial cells, and pericytes induce overexpression of proangiogenic factors in ischemic conditions^22^. In this respect, it is speculated that a region containing more cells will lead to greater metabolic impairment and thus greater clinical impact under ischemic condition.

The distribution of photoreceptors and ganglion cells varied with eccentricity. About 50% of total cones are within 18° of the foveola and about 50% of total ganglion cells are within 13° of the foveola^23^. Additionally, the central foveola, within 1.25° (350 μm in diameter), is free of rods. The density of rods increases most rapidly superiorly and least rapidly nasally from the fovea, and the highest at 20° from the foveal center^17^. Because of the much greater number of cones and rods than image pixels and its uneven distribution, the weighted ISI reflecting differences of regional metabolic activity might be lower than the non-weighted ISI regardless of the retinal zone or cell type. In this study, the difference between the non-weighted and weighted ISI was greatest in the posterior region. This result suggests that non-weighted ISI may be overestimated in the posterior pole, which may lead to limitations in analyzing the association with clinical features. Therefore, there might be a great benefit in the weighted ISI, which differs from the non-weighted ISI in which the pixels of a two-dimensional image are evenly distributed.

In this study, both non-weighted and weighted ISI of NPR with leakage demonstrated a better correlation with clinical features than NPR without leakage. Similarly, Fang et al. reported that the grayish retinal region with leakage on UWF FA was positively correlated with CMT, whereas the entirely dark region without leakage was negatively correlated with CMT^24^. They suggested that the grayish region with leakage might have more viable cells that could produce cytokines resulting in ME. Thus, the results of this study can be explained by a better correlation with NPR with leakage, including cell dysfunction, rather than complete cell death.

Several studies demonstrated the effects of ischemic insults on the retina^2,3,11–13^. Histologically, a reduction of inner retinal thickness occurred before the reduction of outer retinal thickness in rats, suggesting that photoreceptors are less susceptible^11^. However, most studies investigated the impact of ischemia on the inner retina; a few studies focused on photoreceptors^11–13,25^. Recent studies demonstrated reductions in the overall retinal thickness and the loss of retinal ganglion cells, glial cells, and photoreceptors after ischemia induction, suggesting degradation of all retinal layers^11^. Therefore, it appears reasonable to consider changes of metabolism and impairment of ganglion cells and photoreceptors in long-lasting ischemic condition. Although several studies suggested that retinal ganglion cells are the most susceptible to ischemic damage^11–13,12^, the ganglion cell weighted ISI did not show any correlations with clinical features in this study. These results might be related to the lower density of ganglion cells than of cones and rods. The different distributions, densities, and energy requirements of cells could elucidate why the rod weighted ISI had more correlations with clinical findings. Furthermore, a rod-derived cone viability factor causes a metabolic interaction between rods and cones, suggesting that rod damage causes the extinction of intact cones^26,27^.

No consistent association between NPR and DME has been identified. Wessel et al. found an association between the binary classification of peripheral ischemia and the presence of DME^6^. However, they did not find a correlation between the degree of ischemia and CMT. In contrast, Fan et al. reported that NPR in the mid-periphery was significantly correlated with CMT^28^. In this study, the presence of ME was not associated with the non-weighted ISI of NPR with leakage, while it had a positive association with the weighted ISI of NPR with leakage. It is speculated that because the weighted ISI may reflect the cell distribution containing metabolic activity, it showed a better correlation. Previous studies demonstrated that NPR larger than 23% of the total visible retina on UWF FA was more strongly correlated with posterior segment NV^8–10^. This study adds the finding that the presence of baseline NV had stronger correlations with the photoreceptor-weighted (particularly rod-weighted) ISI of NPR with leakage, while it did not correlate with the non-weighted ISI.

Various cytokines are considered as the main mechanism driving the pathological processes like DME or NV. Previous studies showed elevated levels of MCP-1, IL-8, and IL-6 in aqueous humor of DR eyes, which might affect intercellular gap junctions and intracellular tight junctions, thereby increasing vascular permeability^29–32^. PlGF and VEGF are involved in angiogenesis and inflammatory process through the enhancement of tissue factor production and chemotaxis^33^. In this study, the weighted ISI of NPR with leakage was correlated with the levels of MCP-1, IL-8, IL-6, PlGF, and VEGF-A. However, the non-weighted ISI of NPR with leakage was correlated only with the levels of IL-8 and IL-6. These results suggest that increased cytokines and quantification considering metabolic changes may better reflect pathological processes. The correlation between weighted ISI and cytokine level was maintained when cone or rod weighted, but not when ganglion cell weighted. Because of the low density of ganglion cells, regional metabolic changes of them might not affect the results.

This study had some limitations. First, the sample size was relatively small because the data were prospectively collected. Second, manual segmentation of the NPR tends to have inter-grader variability. However, the kappa value between graders was 0.92 (range 0.89–0.94), with a high level of repeatability. Third, because this method only overlaid the density maps of cones, rods, and ganglion cells onto UWF FA images, the results might not fully reflect the metabolic activity. The weighted ISI could more closely reflect the metabolic relevance of a pathological condition by integrating more retinal cells, such as retinal pigment epithelial cells or glial cells, which could show biological activity. Fourth, it is known that there is an individual variation of photoreceptors according to the retinal region. Specifically, it has been reported that the peak foveal cone density is highly variable, although the total foveal cone number is similar between individuals^17^. Nevertheless, to our knowledge, this is the first study to analyze the clinical significance of weighted values applied to each pixel through histological mapping in patients with DR, which could be supported by a cytokine assay.

In conclusion, the weighted ISI reflecting both metabolic activity and cell distribution showed a better correlation with clinical features than non-weighted ISI, which was conventionally used in previous studies. Specifically, the weighted ISI was more valuable in NPR with leakage containing more viable tissue than in NPR without leakage. The presence of baseline ME or NV in patients with DR was associated with the weighted ISI, with a stronger association when the cones and rods were weighted. This quantification method reflecting the metabolic activity at the cellular level will provide new insights into the pathogenesis and prediction of clinical features in patients with DR.

## Methods

### Participants and aqueous humor sampling

The study included 32 patients with treatment-naïve DR; all were aged ≥ 19 years. Images were excluded if there were indistinguishable NPR or NV because of poor image quality or media opacity. Eyes with other retinal diseases (age-related macular degeneration, vascular disease, or uveitis) or spherical equivalent of the above 3-diopters were excluded. The control group comprised 21 age-matched participants with no systemic or ocular diseases who were scheduled for cataract surgery. Aqueous samples were obtained just before anti-VEGF injection or cataract surgery by anterior chamber paracentesis (0.1 mL/patient), frozen immediately, and stored at − 80 °C until analysis. Informed consent was obtained from all participants. The study protocol was approved by the Institutional Review Board (IRB) of Yeungnam University Medical Center (IRB No: 2021-12-005) and the study was conducted in accordance with the tenets of the Declaration of Helsinki.

### Ophthalmic examinations

All participants underwent comprehensive ophthalmic examinations including best-corrected visual acuity (BCVA), fundus examination with slit-lamp biomicroscopy, and spectral-domain optical coherence tomography (SD-OCT, Spectralis, Heidelberg Engineering, Heidelberg, Germany). UWF FA (Optos California, Optos, Dunfermline, UK) was performed in patients with DR. Central macular thickness (CMT) was measured by OCT; it was defined as the mean thickness in the central 1000 μm diameter area. The presence of ME was defined as abnormal retinal thickening with increased fluid volume in the macula.

### Measuring cytokines in aqueous samples

Cytokine levels were quantified using a suspension array (xMAP, Luminex, Austin, TX, USA). Cytokines (angiopoietin [Ang]-1, Ang-2, monocyte chemoattractant protein [MCP]-1, interleukin [IL]-6, IL-8, platelet-derived growth factor [PDGF]-AA, placental growth factor [PlGF], and VEGF-A) were also detected using capture bead kits (Beadlyte, Upstate Biotechnology, Lake Placid, NY, USA). Cytokine levels were calculated from standard curves of each cytokine, using the Master Plex QT 2010 software (Miraibio, Hitachi, CA, USA).

### Metabolic quantification using UWF FA

Early to mid-phase UWF FA (between 40 s and 2 min after injection, mean image acquisition time was 92.1 ± 10.7 s) images were transformed using stereographic projection to correct peripheral distortion. The total visible retina and NPR were outlined by two retinal specialists (M.S. and A.J.) using ImageJ software (version 1.53 h; National Institutes of Health, Bethesda, MD, USA, http://imageJ.nih.gov/ij). NPR was subclassified by the presence or absence of leakage. NPR without leakage was defined as a homogeneously dark, black region caused by capillary loss. NPR with leakage was defined as the region containing both the gray region with leakage, which was from larger vessels (dilated capillaries and microaneurysms, but still hypofluorescent compared to the surrounding area) and NPR without leakage. After binarization, the masked NPR was converted to square millimeters (mm^2^).

**Figure 3:**
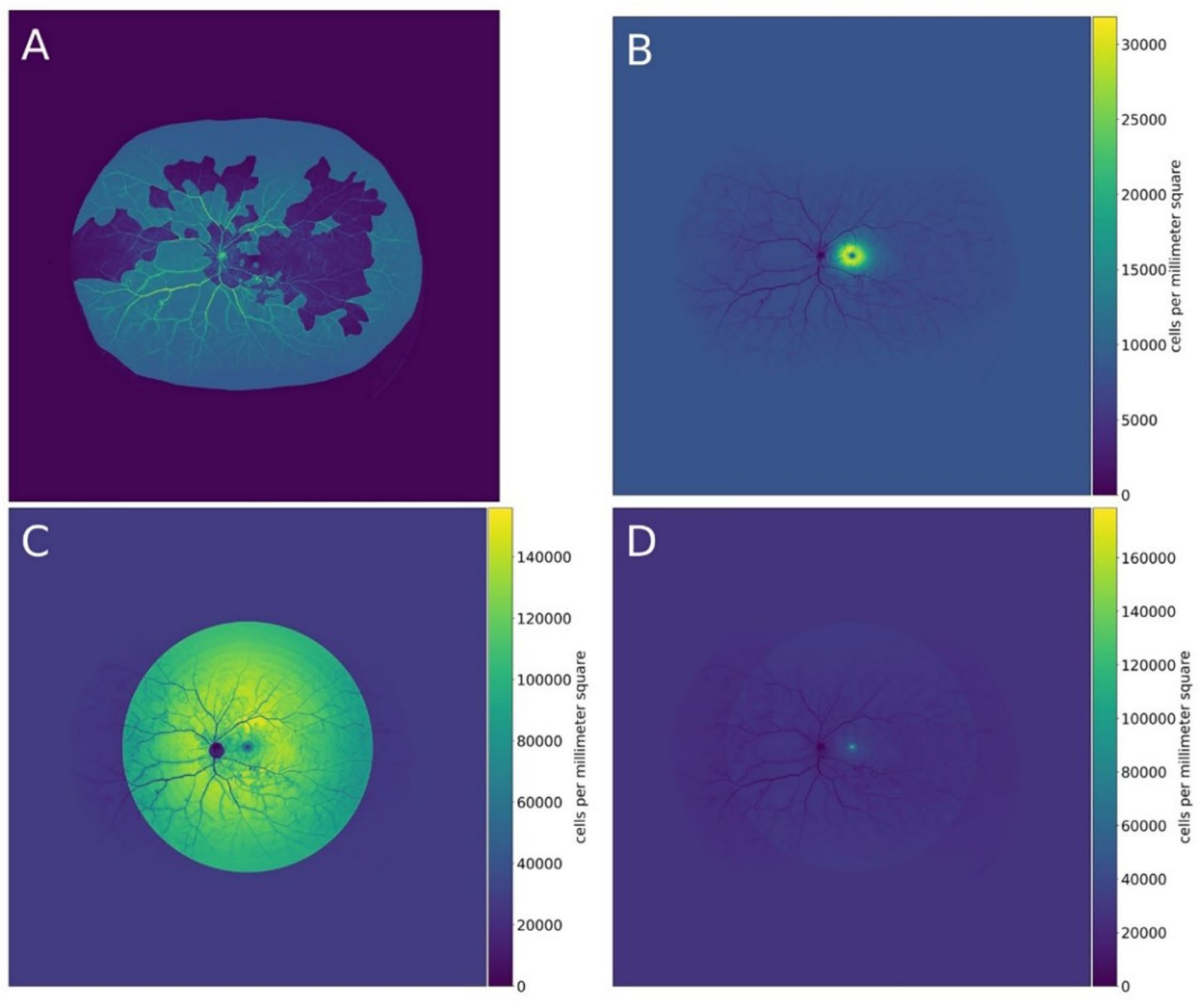
Metabolic quantification of ultra-widefield fluorescein angiography (UWF FA) image. (**A**) Shows an UWF FA image with annotation of the nonperfusion region with leakage overlaid in green. (**B**) Shows the ganglion cell density map overlayed on the same UWF FA image with a density scale in cells per square millimeter. The estimated number of affected ganglion cells of 1 pixel is calculated by multiplying the size of that pixel on the retina in square millimeters with the ganglion cell density of the corresponding pixel in (**B**) in cells per square millimeters. The total number of affected ganglion cells in the annotated area (green) in (**A**) is the sum of these multiplications for all pixel parts of the annotated area (green) in (**A**). (**C**) shows the rod photoreceptor density map and (**D**) shows the cone photoreceptor density map overlayed on the same UWF FA image.

For metabolic quantification, the spatial density of cones and rods, as well as ganglion cells, was topographically plotted on to spherical model eye used for stereographic projection. The density map was generated by fitting four Doniach asymmetric curves^18^ to data points from the density map reported by Curcio et al.^16,17^, by counting via microscopy on ex vivo human retina. Then the curves were transposed to the retinal surface of the model eye with a linear interpolation. With this method, the density of cells could be overlaid for every pixel. By combining the topographic mapping of cell density data (cells/mm^2^) and the anatomically correct area of the retina (mm^2^), the total number of cells in selected region can be calculated as area × cell density = total cell number. The ischemic index (ISI) considering the regional distribution of cells was defined as weighted ISI; otherwise, it was defined as a non-weighted ISI which defined as the ratio of the NPR to the total retinal area. The weighted ISI was defined as the ratio of the cell number in the NPR to the cell number in the total retina. A representative case is illustrated in Fig. 3. The NPR of the UWF FA image was annotated (Fig. 3A). Before stereographic projection, the ISI, the ratio of nonperfused pixels to the total pixels of the visible retina was 0.79 (4,812,641 out of 6,107,592 pixels). After projection, the non-weighted ISI was 0.65 (558/865 mm^2^). The ganglion cell weighted ISI was 0.33 (293,381/887,157 cells) (Fig. 3B). The rod weighted ISI was 0.58 (42,567,992/74,067,521 cells) and cone weighted ISI was 0.55 (1,880,985/3,441,219 cells) (Fig. 3C,D). The total weighted ISI was 0.57.

To analyze the regional distributions of the ISI, the images were divided into three zones using two concentric rings centered on the fovea: posterior (within a radius of 10 mm), mid-peripheral (10–15 mm), and far-peripheral (> 15 mm) regions.

### Statistical analysis

All statistical analyses were performed using SPSS 21.0 for Windows (SPSS, Chicago, IL, USA). The Mann–Whitney U test and the chi-squared test were used to compare variables between the DR and the control groups. The ISI between different zones was compared using the Friedman’s test. The association of the ISI with BCVA, CMT, and cytokine levels were analyzed using the Spearman rank correlation. Multiple linear regression analysis was performed with weighted ISI according to cell type as outcomes. *P*-value less than 0.05 was considered statistically significant.

## Data Availability

The datasets generated during and/or analysed during the current study are available from the corresponding author on reasonable request.
